# Investigating the Hemorheological, Metabolic, and Physical Performance Effect of a Core Muscle Strengthening Training Program

**DOI:** 10.3390/life15091438

**Published:** 2025-09-14

**Authors:** Tobias Mody, Zsuzsanna Nemethne Gyurcsik, Csaba Attila Bakos, Bela Horvath, Barbara Bedocs-Barath, Adam Varga, Adam Attila Matrai, Norbert Nemeth, Sandor Szanto

**Affiliations:** 1 Department of Sports Medicine, Faculty of Medicine, University of Debrecen, H-4032 Debrecen, Hungary; mody.tobias@med.unideb.hu (T.M.); gyurcsik.zsuzsanna@med.unideb.hu (Z.N.G.); szanto.sandor@med.unideb.hu (S.S.); 2 Doctoral School of Clinical Medicine, University of Debrecen, H-4032 Debrecen, Hungary; 3 University of Debrecen, H-4002 Debrecen, Hungary; csaba.a.bakos@gmail.com; 4 Armoured Infantry Brigade, Hungarian Defense Forces, Bocskay István 11, H-4031 Debrecen, Hungary; horvath.bela@mil.hu; 5 Department of Operative Techniques and Surgical Research, Faculty of Medicine, University of Debrecen, H-4032 Debrecen, Hungary; barath.barbara@med.unideb.hu (B.B.-B.); varga.adam@med.unideb.hu (A.V.); matrai.adam@med.unideb.hu (A.A.M.)

**Keywords:** physical exercise, performance, core muscle training, hemorheology, metabolites

## Abstract

Physical activity influences red blood cell (RBC) deformability and aggregation, which affect oxygen transport and performance. While regular training may enhance RBC properties, adaptations depend on exercise intensity, duration, and recovery. This study aimed to assess the impact of a 12-week core muscle training program on RBC deformability, aggregation, and aerobic capacity in military trainees. A total of 35 male volunteers were divided into a Training group (n = 17) and a Control group (n = 18). The intervention included dynamic stretching, core stabilization, and functional movement exercises. Spiroergometry tests, blood gas analysis, and hemorheological measurements were conducted before and after the program. Results showed no significant changes in body composition or aerobic capacity. RBC deformability slightly decreased after exercise in both groups, while RBC aggregation increased. Blood viscosity changes were more moderate in the Training group, suggesting potential adaptation. However, the training intensity may have been insufficient for significant hemorheological improvements. While regular physical activity can enhance RBC function, adequate intensity, recovery, and nutrition are essential for optimal adaptation. Individualized training strategies should consider these factors to maximize performance and hemorheological benefits.

## 1. Introduction

One of the most important prerequisites for strong physical activity is adequate oxygen supply to the muscles, which is determined by the maximum cardiac output, gas exchange in the lungs, and the oxygen-carrying capacity of the blood among the central components, and the oxygen absorption capacity of the muscles and the activity of mitochondrial enzymes among the peripheral components [1,2,3,4]. The direct connection between central and peripheral factors is represented by the blood flow in the small vessels of the muscles, which, in addition to vascular factors, is influenced by whole blood viscosity [5].

Whole blood can be characterized as a non-Newtonian fluid, i.e., its viscosity changes with the shear rate [6,7]. The most important factors determining the alteration of whole blood viscosity during physical activity are the ratio of cellular elements, practically the hematocrit, the viscosity of the plasma, which depends on protein—mainly the fibrinogen concentration within it—and the deformability and aggregation of the red blood cells [6,7].

The deformability of red blood cells is crucial in microcirculation, as these cells have to pass through blood vessels that are often smaller than their diameter. Regular exercise can improve the hemorheological status, including the deformability of red blood cells [2,8,9,10,11,12,13,14,15]. According to recently published data, 6 weeks of endurance training is associated with a significant reduction in the shear stress required to achieve half-maximum deformation to theoretical maximal elongation in red blood cells [14]. In accordance with these results, a better deformability of red blood cells was found in endurance athletes compared to athletes involved in team or combat sports [16]. Aggregation of red blood cells is determined by cellular and plasmatic factors, including fibrinogen concentration [7,17]. Various forms of aerobic exercise can also have different effects on the aggregation of red blood cells. In well-trained individuals, cycling increases aggregation as a result of short-term, intense loading, while aggregation does not change during running, but deformability increased [3,9].

Our research group previously investigated the changes in the hemorheological parameters of elite athletes (soccer and ice-hockey players) and control subjects during short-term, high-intensity exercise [15]. The increase in whole blood viscosity due to exercise was smaller among the elite athletes. Red blood cell deformability worsened significantly only in the Control group but was not changed in the elite athletes by the load. The aggregation values were more stable in athletes, especially in ice-hockey players [15].

Basic military training requires improvements in elements of physical fitness, including aerobic and anaerobic endurance, muscle strength, agility, and flexibility. Since the length of the trainings, the time available for physical development is limited, the general health status of young people in countries with high income is becoming weaker, and the initial physical performance of the conscripts differs significantly, the selection of the appropriate methods used for physical development is becoming more and more important [18,19]. In addition to the exercises used in traditional training (such as running, sprinting, push-ups, pull-ups, and squats), new training methods are increasingly gaining ground. In recent years, numerous publications have reported on the beneficial effects of non-traditional training programs on endurance and muscle strength, most of which recommend the inclusion of high-intensity functional training programs in the training process [20,21].

The beneficial effect of functional strengthening exercises as part of military training has recently been published. In this study, the frequency of lower back pain decreased, and the endurance of the lumbar muscles improved as a result of the core muscle strengthening training compared to participants in the traditional training program [22]. Targeted strengthening of this muscle group improves physical performance, such as vertical jumping among athletes, and mitigates biomechanical risk factors that predispose to injury to the anterior cruciate ligament of the knee [23]. More generally, based on a recently published systematic review, core muscle strengthening exercises lasting 4–12 weeks have a beneficial effect on sport-specific skills in many sports, including football, handball, basketball, rowing, karate, volleyball, and badminton [24]. Core-strengthening training enhances venous return and respiratory efficiency through improved trunk stability and diaphragmatic function, thereby supporting cardiovascular performance. These adaptations may contribute to favorable hemorheological changes, including improved red blood cell deformability and reduced blood viscosity, which have been observed following structured resistance training programs [25,26,27].

Based on these findings, incorporating core muscle strengthening into daily training routines is increasingly recommended. Previous research has consistently demonstrated that physical training influences hemorheological parameters, including red blood cell deformability, aggregation, and blood viscosity. Improvements have been reported mainly in endurance athletes, where higher training loads and long-term adaptations support more favorable microcirculatory function. These findings underline the role of regular exercise in maintaining “hemorheological fitness”. However, the impact of non-traditional exercise approaches, such as core strengthening programs, on hemorheological adaptations has not been thoroughly investigated. While core training is widely recognized for its role in musculoskeletal stability, injury prevention, and breathing mechanics, its potential influence on blood rheology remains largely unexplored. Addressing this knowledge gap was the primary motivation for our study.

## 2. Materials and Methods

### 2.1. Volunteers

Thirty-five men participated in our study (ethical permission nr.: DE RKEB/IKEB:5410-2020, University of Debrecen). All of them were volunteer individuals applying for military training program of the Hungarian Defense Forces. The selection was made on a voluntary basis; however, they had to take part in a basic physical examination, which consisted of the following elements: running (Cooper test), sit-ups, pull-ups, push-ups.

The participants were then randomly divided into two groups. Participants were randomly assigned to the Training or Control group. A computer-generated random number sequence (block randomization; block size = 4) was prepared by an independent researcher not otherwise involved in the study. Neither the trainers nor the participants could influence the randomization process. The research team responsible for outcome assessment was, whenever possible, blinded to group allocation. After the first surveys, the participants of the Training group (17 men; age: 23.47 ± 5.92 year; height: 180.82 ± 9.14 cm) took part in a 3-month core muscle developing training session beyond the basic military training program. The Control group participants (18 men; age: 22.88 ± 5.44 year; height: 180.28 ± 8.56 cm) were not involved in any special training besides basic military training.

### 2.2. The Core Muscle Training Program

The duration of the intervention was 1 h per day for 12 weeks. The aim of the intervention was to improve general flexibility and to improve the activity and functional synergies of the core muscles. The following factors were taken into account when compiling the exercise program:

Dynamic stretching in standing and crawling positions, which included muscle chains and their synergist activity. Dynamic stretching is the most effective method for warming up because it has a positive effect not only on joint range of motion (ROM) but also on muscle strength, power, and explosiveness, and it also improves proprioception and coordination. After a dynamic warm-up, greater joint ROM is explained by a decrease in the tension of the muscle-tendon transition. The improvement in performance can be attributed to increased muscle temperature due to active, repeated stretching and contraction through decreased viscous muscle resistance. During dynamic stretching, the movement is carried out over the entire range of motion; when the agonist muscle contracts, the antagonist muscle relaxes, but the end position is not maintained, and the muscle is in the stretched state for less time than when performing static stretching. Rotational mobilizing exercises combine with breathing control to reduce the tone of tight muscles. The general objective of rotation is to reduce the increased muscle tone and stiffness. This could improve the power transmission role of the elastic muscles.

Chest mobilization, which contributes to optimal breathing techniques and increases the core stabilizing function. Since the diaphragm forms the superior wall of the inner box of the core in the local stabilizer group, it also contributes to segmental stabilization, ensuring the deep muscle synergist function in the horizontal plane. The local stabilizers contain the deep muscle box (multifidus and transvers abdominal muscles, muscle of pelvic floor, diaphragm). The global stabilizers control movements in the frontal plane, while the global mobilizers contribute to concentric and eccentric activities in the sagittal plane.

Targeted core strengthening using different crawling position heights in static and progressive form. Between high and deep crawling positions, which could improve muscle chain activities, we can achieve stability and plasticity of the spine through the dynamic work of the muscles; moreover, the unloaded starting position reduces the compressive force acting on the elements of the movement segments and protects the muscles from overload. We adapted the Functional Movement Screen Test positions in practice, breaking them down into elements to improve the general movement patterns [28,29,30,31].

The description and the detailed tasks of the core muscle training program are summarized in Tables S1 and S2.

### 2.3. Body Composition, Spiroergometry Test, and Collection of Blood Samples

Participants started with body composition examination by InBody 770 (InBody USA Co., Ltd., Cerritos, CA, USA) to define tissue compositions per body region. Immediately after this, venous blood samples were obtained (median cubital vein, 23 G needle, Vacutainer tubes K_3_-EDTA) for laboratory tests. Ten minutes later, all participants undertook a spiroergometry test on a treadmill ergometer. We used a ramp protocol (Vitamaxima 12) with increasing speed and elevation; the load was increased by 45 W per minute. The maximal velocity was 12 km/h, and the elevation was increased to a maximum of 17.5 degrees [32,33]. The average maximal workload was 345.00 ± 36.46 W.

Gas exchange parameters were detected by Vyntus CPX hardver (Vyaire medical, Mettawa, IL, USA). Heart rate was measured by a Polar H9 (Polar Electro, Kempele, Finland) chest belt, and real-time data were trackable using a Bluetooth connection. The test was performed at a temperature of 20 °C. Exercise was stopped when participants reached their maximal performance capacity. Recorded main parameters were load [W], time [min], heart rate [1/min], ventilation volume [L/min], breathing frequency [1/min], oxygen uptake as volume (VO_2_ [mL/min]), carbon dioxide output as volume (VCO_2_, [mL/min]), respiratory exchange ratio (RER, VCO_2_/VO_2_), and systolic and diastolic blood pressure [mmHg]. To ensure comparability with other studies, VO_2_max determination was based on direct breath-by-breath gas exchange analysis using a face mask connected to the metabolic cart. This allowed us to measure the absolute volume of oxygen uptake throughout the test. In all participants, oxygen consumption reached a plateau despite the increasing workload, which confirmed that a true maximal oxygen uptake value had been achieved.

Right after the exercise, we collected venous blood samples again and measured the heart rate for 5 additional minutes. In this 5 min resting period, we also collected capillary blood samples from the participants’ fingertips (right hand, index finger with Accu-Chek Safe T-Pro lancets) to determine the blood lactate concentration in the first and fifth minutes of resting. To test lactate concentration [mmol/L], a Nova Lactate Plus device was used (Nova Biomedical, Waltham, MA, USA).

### 2.4. Laboratory Blood Tests

Hematological variables (general quantitative and qualitative parameters) were determined using a Sysmex K-4500 automated system (TOA Medicor Electronics Co., Ltd., Komatsu City, Japan).

Through an EPOC^®^ Blood Analysis System (Siemens Healthineers AG, Erlangen, Germany), the partial oxygen and partial carbon dioxide pressures (pO_2_, pCO_2_ [mmHg]), pH, and lactate [mmol/L], creatinine [μmol/L], and glucose [mmol/L] concentrations were analyzed.

Whole blood viscosity (WBV) and plasma viscosity (PV) were measured by a Hevimet-40 capillary viscometer (Hemorex Ltd., Budapest, Hungary). For comparison, blood viscosity values at 90 s^−1^, corrected WBV values for 40% hematocrit (using the Matrai-formula [34]), and Hct/WBV ratio data were used.

A LoRRca MaxSis Osmoscan ektacytometer (Mechatronics BV, Zwaag, The Netherlands) was used to assess RBC deformability [35,36]. A 10 µL blood sample was diluted in 2 mL of a polyvinyl–pyrrolidone (PVP)/phosphate-buffered saline (PBS) solution (viscosity: 25.2–26.4 mPas; osmolarity: 290–310 mOsm/kg; and pH: 7.2) for the tests. All measurements were carried out at 37 °C [36]. Red blood cell elongation index (EI) values were determined in the function of shear stress (SS [Pa] at a range of 0.3–30 Pa. Individual EI-SS curves were compared using the EI values at 3 Pa, as well as the maximal elongation index (EI_max_), the shear stress at half EI_max_ (SS_1/2_, [Pa]), and their ratio (EI_max_/SS_1/2_), calculated via the Lineweaver–Burk equation [37].

Red blood cell aggregation was investigated using light-transmission (Myrenne MA-1 aggregometer, Myrenne GmbH, Roetgen, Germany) and light-reflection methods (LoRRca MaxSis Osmoscan ektacytometer, Mechatronics BV, Zwaag, The Netherlands) [35,36]. Four aggregation parameters were determined by the Myrenne MA-1: aggregation M index values under stasis (M 5 s, M 10 s) and M1 values at 3 s^−1^ shear rate (M1 5 s, M1 10 s). The measurements were taken at room temperature (20–25 °C). Each index parameter was determined using four parallel measurements, the average of which we used. Further parameters could be determined in the LoRRca MaxSis Osmoscan ektacytometer (Mechatronics BV, Zwaag, The Netherlands) using the laser backscattering method. The blood sample (1 mL) was disaggregated by rotation in the Couette system, and when the rotor stopped, the aggregation process began, while a syllectogarm of the reflected laser beam (intensity) was analyzed. This device determines the aggregation index (AI [%]), the amplitude (Amp [au]), and the half-time of intensity curve (aggregation half time, t1/2 [s],) [35,36].

### 2.5. Statistical Analysis

A SigmaStat Software 3.1.1.0. (Systat Software Inc., San Jose, CA, USA) was used for statistical analyses. Data are presented as means ± standard deviation (S.D.). Depending on the normality of data distribution, t-test or the Mann–Whitney rank-sum was used for inter-group comparison, and one-way and repeated-measure ANOVA or Kruskal–Wallis’s tests were used for intra-group comparison, with a significance level of *p* < 0.05.

## 3. Results

### 3.1. Bodyweight, Body Mass Index, and Body Composition Parameters

Table 1 summarizes the weight, body mass index (BMI), and body composition data of the participants before and after the 3-month program.

Although no statistically significant changes were observed, the Training group demonstrated a slight reduction in body fat percentage, suggesting the early onset of favorable body composition adaptations, even within a relatively short intervention period.

### 3.2. Spiroergometry Test

Respiratory exchange rate (RER) slightly increased by the end of the 3-month period, without significant difference. During the spiroergometry test, maximal lactate concentration demonstrated only a moderate overall decrease; however, the Training group exhibited a clearer tendency toward faster lactate reduction by the end of the program. This trend may reflect an improved ability to manage metabolic by-products during recovery, even in the absence of significant group differences. (Table 2).

### 3.3. Blood Gas, pH, Acid–Base Parameters, Electrolytes, and Metabolites

During spiroergometry testing, intensive physical activity is required; therefore, an increase in *p*O_2_, a decrease in *p*CO_2_, a decrease in pH, and an increase in metabolite concentrations were observed, as expected. Blood gas parameters showed similar alterations in both groups: at the end of the 3-month period, the magnitude of the alterations decreased. There were no significant differences in pH decrease or lactate concentration increase between the groups. Creatinine concentration between both groups just after the spiroergometry test started from higher values at the end of the 3-month period (Table 3).

### 3.4. Hematological and Hemorheological Parameters

In both groups, white blood cell and platelet counts increased similarly and significantly after the spiroergometry test. The same alterations were observed at the end of the 3-month period. Red blood cell counts and hematocrit values reflected the moderate hemoconcentration just after the spiroergometry test (Table 4).

The increase in whole blood viscosity after spiroergometry testing was much more moderate at the end of the 3-month period, and in the Training group, an acute increase was observed. The blood viscosity values corrected by 40% hematocrit showed a significant decrease in the Training group, with a higher hematocrit to blood viscosity ratio (Table 5).

**Figure 1:**
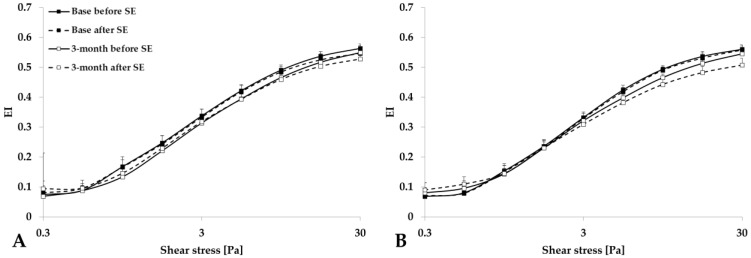
Elongation index (EI)–shear stress (SS) curves for the Control group (**A**) and Training group (**B**), tested before and after spiroergometry (SE), with test conducted at the beginning of the program (base) and at the end of the 3-month program. Means ± S.D.

**Figure 2:**
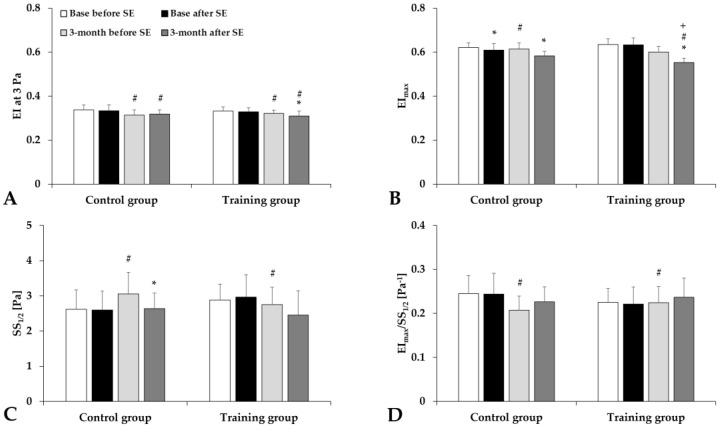
Elongation index (EI) at 3 Pa (**A**), EI_max_ (**B**), shear stress at half EI_max_ (SS_1/2_) (**C**), and their ratio (**D**) in the Control and Training groups, tested before and after spiroergometry (SE), with tests conducted at the beginning of the program (base) and at the end of the 3-month program. Means ± S.D.; * *p* < 0.05 vs. before spiroergometry; # *p* < 0.05 vs. base, + *p* < 0.05 vs. Control.

Analyzing the elongation index–shear stress curves, we found that red blood cell deformability slightly decreased just after the spiroergometry test in both groups (Figure 1). The elongation index at 3 Pa significantly decreased, the maximal elongation index moderately decreased, and the SS_1/2_ values slightly increased or decreased (Figure 2).

**Figure 3:**
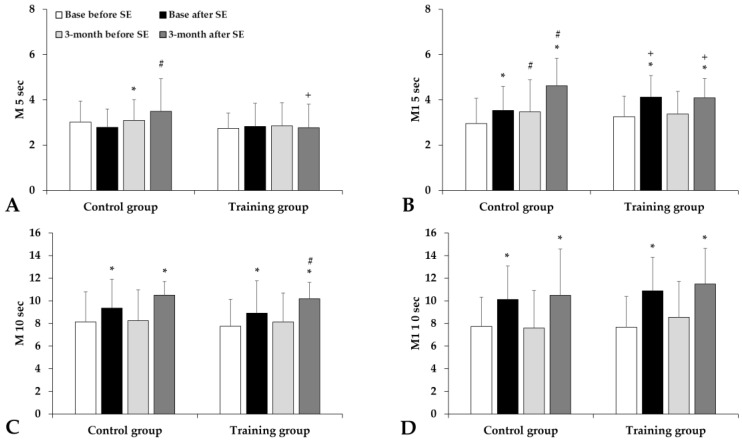
Aggregation index values by light-transmission method: M 5 sec (**A**), M1 5 sec (**B**), M 10 sec (**C**), and M1 10 sec (**D**) in the Control and Training groups, tested before and after spiroergometry (SE), with tests conducted at the beginning of the program (base) and at the end of the 3-month program. Means ± S.D.; * *p* < 0.05 vs. before spiroergometry; # *p* < 0.05 vs. base, + *p* < 0.05 vs. Control.

**Figure 4:**
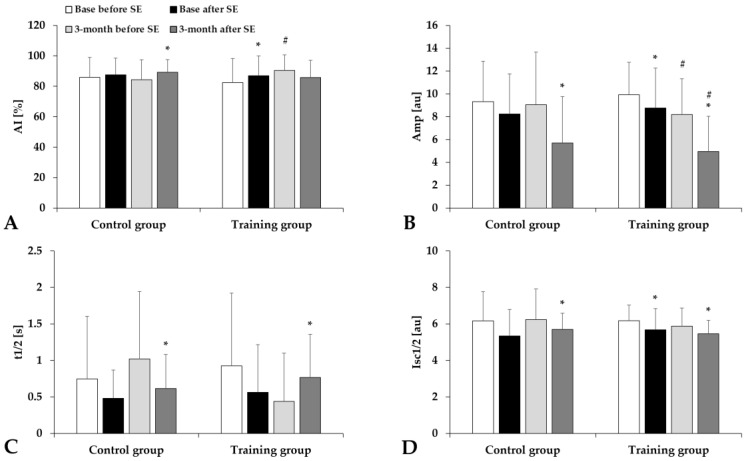
Aggregation index values by syllectometry method: AI (**A**), Amp (**B**), t1/2 (**C**), and Isc1/2 (**D**) in the Control and Training groups, tested before and after spiroergometry (SE), with tests conducted at the beginning of the program (base) and at the end of the 3-month program. Means ± S.D.; * *p* < 0.05 vs. before spiroergometry, # *p* < 0.05 vs. base.

The red blood cell aggregation index (M index values) significantly increased without significant difference between the groups (Figure 3). The aggregation index in syllectometry slightly increased, and the amplitude values and the t1/2 values decreased in the Training group (Figure 4).

## 4. Discussion

The impact of physical activity on red blood cell (RBC) deformability and aggregation has been extensively studied, revealing nuanced effects based on exercise intensity, duration, and the individual’s training status. Research indicates that while acute, high-intensity exercise can lead to increased RBC aggregation, RBC deformability often remains unchanged. In a 6-week resistance training study (70% vs. 85% 1RM), RBC aggregation rose significantly immediately after each session in both groups, whereas RBC deformability did not change significantly in the heavier (85% 1RM) condition (it increased only in the moderate-intensity group on day 1) [27].

A study involving cyclists demonstrated that despite a significant rise in blood lactate levels during heavy-intensity exercise, red blood cell deformability did not exhibit notable alterations [38]. The changes in erythrocyte deformability observed during our investigation, as well as the alterations measured after load, can be attributed to the following explanations: Enhancing red blood cell deformability, which positively impacts oxygen transport, microcirculation, and, ultimately, physical performance, requires the adaptive processes of red blood cells in response to stress [2,11,14,15,38,39]. However, the type, duration, intensity, and regularity of exercise influence and determine whether the resulting stress can trigger the appropriate adaptation mechanisms from a hemorheological perspective. Among these stress factors are oxidative stress, which becomes more pronounced at higher intensities during physical activity, and mechanical stress on red blood cells, known as shear stress, associated with increased circulation. As a result of these influences, red blood cells can modify the structure of their membrane proteins through various physiological adaptation mechanisms, leading to improved deformability [7,15,38,39].

In our study, despite the relatively long intervention period, the training intensity appeared insufficient to induce measurable improvements in RBC deformability. This was confirmed by the results of the spiroergometry tests, in which we did not perceive any significant positive changes in terms of performance and endurance (VO_2_max) after the intervention period.

Alongside the type of exercise, proper recovery and nutrition also play a vital role in the adaptation of red blood cells, particularly in maintaining deformability and membrane stability. The flexibility and durability of the RBC membrane rely on structural proteins like spectrin, actin, and ankyrin, which require adequate protein synthesis supported by a balanced diet [40]. Antioxidants such as vitamins C and E and polyphenols protect RBC membranes from oxidative damage caused by intense physical activity [41]. Sufficient hydration prevents increased plasma osmolarity and RBC rigidity, which can compromise deformability [42]. Additionally, micronutrients like iron, vitamin B12, and folate are essential for RBC formation and function, as deficiencies in these nutrients are associated with impaired erythropoiesis and membrane integrity [43]. Overall, a combination of balanced nutrition, proper rest, and hydration ensures optimal RBC performance during and after exercise. This study has only a few limitations: First, the sample included only male participants, which restricts the generalizability of the findings. Second, nutrition and recovery were not controlled; unlike professional athletes, participants did not follow a personalized diet or receive nutritional supplementation. In addition, the extra core training sessions may have reduced recovery time, limiting processes such as RBC membrane stabilization that support deformability [44]. These factors may partly explain the absence of detectable hemorheological adaptations. Nonetheless, the study provides novel insights into the potential role of core training within military physical preparation. Considering the limitations of the present study, future investigations should incorporate standardized and mandatory elements of nutrition and recovery protocols and may also consider increasing the training intensity in the active group to achieve more significant hemorheological adaptations and performance outcomes.

## 5. Summary and Conclusions

These observations suggest that regular, sport-specific training may confer adaptations that preserve or enhance “hemorheological fitness”, even under the stress of high-intensity exercise. The mechanisms underlying these adaptations could involve improved oxidative stress management, alterations in plasma composition, and enhanced cardiovascular efficiency. Conversely, untrained individuals may experience more significant impairments in RBC deformability and greater increases in aggregation due to a lack of adaptations.

Although the 12-week core muscle strengthening training did not significantly improve aerobic capacity or red blood cell deformability, the training program showed small but measurable changes in other physiological parameters. Participants in the training group showed a modest trend toward reduced body fat percentage and an improved lactate clearance profile during recovery, suggesting potential benefits for metabolic efficiency. In addition, the acute increase in whole blood viscosity was more moderate after the intervention, indicating that core training may exert subtle protective effects on hemorheological responses to exercise. These findings highlight the practical value of incorporating core training into military physical preparation, not as a substitute for endurance conditioning, but as a complementary element that may support recovery and microcirculatory adaptation.

While acute high-intensity exercise can transiently affect RBC properties, regular training, especially when sport-specific, seems to mitigate adverse changes, promoting a hemorheological profile conducive to efficient oxygen delivery and performance. These findings emphasize the need for individualized training approaches that consider the unique hemorheological demands of different sports and the baseline fitness levels of athletes.

## Figures and Tables

**Table 1:** Bodyweight, body mass index (BMI), and further body composition data (tested with Inbody 770 device; InBody USA Co., Ltd., Cerritos, CA, USA) of the participants.

Variables	Control Group		Training Group	
	Base	End of 3-Month Period	Base	End of 3-Month Period
Bodyweight [kg]	83.31 ± 13.15	84.01 ± 13.22	83.78 ± 12.02	83.23 ± 10.90
BMI [kg/m^2^]	25.48 ± 2.93	25.73 ± 2.71	25.59 ± 2.82	25.41 ± 2.18
Percent body fat [%]	16.37 ± 5.89	15.86 ± 5.13	16.06 ± 6.34	14.36 ± 5.43
Skeletal muscle mass [kg]	39.82 ± 5.53	40.49 ± 6.08	40.35 ± 6.57	40.89 ± 6.13

Means ± S.D.

**Table 2:** Respiratory exchange rate (RER), lactate concentration (maximal and 5 min, and their ratio), and their correlation coefficients in Control and Training groups before and after the standardized physical exercise by spiroergometry.

Variables	Control Group		Training Group	
	Base	End of 3-Month Period	Base	End of 3-Month Period
RER	1.11 ± 0.07	1.14 ± 0.09	1.13 ± 0.06	1.17 ± 0.07
Lactate_max_ [mmol/L]	11.30 ± 2.22	10.61 ± 2.49	12.60 ± 1.95	11.74 ± 2.46
Lactate_5’_ [mmol/L]	10.89 ± 2.05	10.23 ± 2.34	12.02 ± 2.11	10.25 ± 2.00
Lactate_max_/Lactate_5’_	1.06 ± 0.22	1.05 ± 0.16	1.06 ± 0.09	1.17 ± 0.28
Lactate decrease in 5 min (%)	2 ± 19%	2 ± 18%	4 ± 9%	11 ± 14%
R^2^ of RER and Lactate_5’_	0.4707	0.2199	0.2716	0.0503

Means ± S.D.

**Table 3:** Changes in blood gas parameters, pH, lactate, creatinine, and glucose concentrations in the Control and Training groups tested before and after spiroergometry tests conducted at the beginning of the program (base) and at the end of the 3-month program.

Variables	Group	Base			End of 3-Month Period		
		Before Spiroergometry	After Spiroergometry	After/Before Ratio	Before Spiroergometry	After Spiroergometry	After/Before Ratio
*p*O_2_ [mmHg]	Control	30.62 ± 10.34	68.97 ± 26.29 *	2.43 ± 1.12	31.15 ± 8.24	54.85 ± 17.95 *	1.91 ± 0.80
	Training	28.78 ± 7.73	70.29 ± 23.41 *	2.56 ± 1.03	32.56 ± 5.43	52.51 ± 17.42 *	1.66 ± 0.64 #
*p*CO_2_ [mmHg]	Control	53.92 ± 5.36	43.60 ± 7.50 *	0.81 ± 0.14	52.01 ± 4.37	48.39 ± 7.21 #	0.93 ± 0.19 #
	Training	54.70 ± 4.01	42.37 ± 7.54 * +	0.78 ± 0.13	55.90 ± 4.58 # +	49.76 ± 6.66 * #	0.89 ± 0.11 #
pH	Control	7.34 ± 0.03	7.23 ± 0.06 *	0.98 ± 0.01	7.34 ± 0.04	7.21 ± 0.05 *	0.98 ± 0.01
	Training	7.34 ± 0.02	7.22 ± 0.07 *	0.98 ± 0.01	7.33 ± 0.02	7.22 ± 0.06 *	0.99 ± 0.01
Lactate [mmol/L]	Control	1.41 ± 0.50	12.57 ± 1.88 *	9.82 ± 3.24	1.74 ± 1.83	13.38 ± 2.99 *	10.40 ± 5.19
	Training	1.35 ± 0.29	13.87 ± 2.42 *	10.65 ± 2.97	1.76 ± 0.52	13.43 ± 3.43 *	8.48 ± 3.65
Creatinine [µmol/L]	Control	84.75 ± 16.75	94.42 ± 12.02 *	1.08 ± 0.30	107.67 ± 19.28 #	121.25 ± 19.01 * #	1.10 ± 0.12
	Training	93.84 ± 21.67	112.47 ± 23.3 * +	1.26 ± 0.46	104.59 ± 14.49 #	116.47 ± 12.76 *	1.12 ± 0.10
Glucose [mmol/L]	Control	5.24 ± 0.58	6.57 ± 1.26 *	1.26 ± 0.23	4.94 ± 0.49 #	5.96 ± 1.37 *	1.20 ± 0.25
	Training	5.00 ± 0.55	6.48 ± 1.24 *	1.32 ± 0.31	4.44 ± 0.56 # +	5.55 ± 0.95 * #	1.29 ± 0.38

Means ± S.D.; * *p* < 0.05 vs. before spiroergometry, # *p* < 0.05 vs. base, + *p* < 0.05 vs. Control.

**Table 4:** Changes in white blood cell count (WBC), red blood cell count (RBC), hemoglobin concentration (Hgb), hematocrit (Hct), mean corpuscular volume (MCV), mean corpuscular hemoglobin (MCH), mean corpuscular hemoglobin concentration (MCHC), and platelet count (Plt) in the Control and Training groups, tested before and after spiroergometry, with tests conducted at the beginning of the program (base) and at the end of the 3-month program.

Variables	Group	Base			End of 3-Month Period		
		Before Spiroergometry	After Spiroergometry	After/Before Ratio	Before Spiroergometry	After Spiroergometry	After/Before Ratio
WBC [×10^9^/L]	Control	6.29 ± 1.17	12.67 ± 2.67 *	2.06 ± 0.47	6.73 ± 1.15	11.37 ± 1.94 *	1.71 ± 0.24 #
	Training	5.82 ± 1.26	11.11 ± 3.22 *	1.92 ± 0.43	5.51 ± 0.99	10.31 ± 2.20 *	1.91 ± 0.51
RBC [×10^12^/L]	Control	5.05 ± 0.28	5.29 ± 0.37	1.05 ± 0.05	5.09 ± 0.36	5.32 ± 0.31	1.06 ± 0.04
	Training	5.20 ± 0.30	5.36 ± 0.32	1.03 ± 0.02	5.15 ± 0.27	5.35 ± 0.26	1.04 ± 0.04
Hgb [g/L]	Control	15.27 ± 0.80	16.05 ± 1.02 *	1.05 ± 0.04	15.28 ± 1.00	16.09 ± 0.90 *	1.06 ± 0.03
	Training	15.82 ± 0.99	16.41 ± 1.04 *	1.04 ± 0.02	15.53 ± 1.06	16.21 ± 0.89 *	1.05 ± 0.05
Hct [%]	Control	44.22 ± 1.82	46.96 ± 2.50 *	1.06 ± 0.05	44.08 ± 2.60	47.34 ± 1.83 *	1.08 ± 0.03
	Training	45.45 ± 2.15	47.57 ± 2.61 *	1.05 ± 0.02	45.27 ± 2.47	48.03 ± 2.25 *	1.06 ± 0.04 +
MCV [fL]	Control	87.71 ± 3.33	88.96 ± 3.61	1.01 ± 0.01	86.66 ± 3.09	89.06 ± 3.49	1.03 ± 0.03
	Training	87.51 ± 2.74	88.80 ± 2.93	1.01 ± 0.01	87.99 ± 2.80	89.84 ± 2.77	1.02 ± 0.08 +
MCH [pg]	Control	30.28 ± 1.20	30.39 ± 1.22	1.00 ± 0.01	30.04 ± 1.31	30.27 ± 1.38	1.01 ± 0.02
	Training	30.46 ± 1.19	30.63 ± 1.19	1.01 ± 0.01	30.18 ± 1.22	30.34 ± 1.23	1.01 ± 0.02
MCHC [g/L]	Control	34.52 ± 0.79	34.17 ± 0.95	0.99 ± 0.01	34.67 ± 0.89	33.98 ± 1.00	0.98 ± 0.02
	Training	34.80 ± 0.92	34.50 ± 0.90	0.99 ± 0.01	34.29 ± 0.79	33.76 ± 0.87	0.98 ± 0.02
Plt [×10^9^/L]	Control	254.80 ± 47.62	318.18 ± 42.46 *	1.29 ± 0.34	258.17 ± 48.91	322.79 ± 42.55 *	1.26 ± 0.18
	Training	217.47 ± 39.69	279.08 ± 66.84 *	1.27 ± 0.12	219.85 ± 40.28	269.35 ± 50.35 *	1.23 ± 0.13

Means ± S.D.; * *p* < 0.05 vs. before spiroergometry, # *p* < 0.05 vs. base, + *p* < 0.05 vs. Control.

**Table 5:** Changes in whole blood viscosity (WBV), plasma viscosity (PV), corrected WBC for 40% hematocrit (Hct) values, and hematocrit-to-blood viscosity ratio in the Control and Training groups, tested before and after spiroergometry, with tests conducted at the beginning of the program (base) and at the end of the 3-month program.

Variables	Group	Base			End of 3-Month Period		
		Before Spiroergometry	After Spiroergometry	After/Before Ratio	Before Spiroergometry	After Spiroergometry	After/Before Ratio
WBV [mPas]	Control	5.03 ± 0.68	5.10 ± 0.56	1.02 ± 0.14	4.57 ± 0.52	5.07 ± 0.46 *	1.11 ± 0.11
	Training	4.70 ± 0.68	5.50 ± 0.64 *	1.18 ± 0.15	5.04 ± 1.01	4.98 ± 0.61 #	1.02 ± 0.20
PV [mPas]	Control	1.45 ± 0.26	1.45 ± 0.34	1.02 ± 0.27	1.36 ± 0.27	1.63 ± 0.45 *	1.21 ± 0.31
	Training	1.45 ± 0.40	1.63 ± 0.27	1.10 ± 0.38	1.49 ± 0.48	1.66 ± 0.40	1.19 ± 0.41
WBV 40% [mPas]	Control	4.41 ± 0.55	4.21 ± 0.46	0.97 ± 0.14	4.11 ± 0.55	4.21 ± 0.33	1.02 ± 0.09
	Training	4.07 ± 0.48	4.55 ± 0.43 *	1.07 ± 0.29	4.37 ± 0.84	4.11 ± 0.30 #	0.97 ± 0.19
Hct/WBV	Control	9.04 ± 1.02	9.30 ± 0.88	1.04 ± 0.18	9.76 ± 1.25	9.40 ± 0.78	0.99 ± 0.10
	Training	9.82 ± 1.21	8.73 ± 0.88 *	0.90 ± 0.12	9.25 ± 1.56	9.73 ± 0.79 #	1.09 ± 0.29

Means ± S.D.; * *p* < 0.05 vs. before spiroergometry, # *p* < 0.05 vs. base.

## Data Availability

The data presented in this study are available on request from the corresponding author. The data are not publicly available due to ethical constraints.

## Supplementary Materials

The following supporting information can be downloaded at https://www.mdpi.com/article/10.3390/life15091438/s1: Table S1: Description of the core training program; Table S2: Detailed tasks of the core training program.
