# Supplementary material for: Investigating the Hemorheological, Metabolic, and Physical Performance Effect of a Core Muscle Strengthening Training Program
Source: Life (Basel). 2025 Sep 14;15(9):1438. doi: 10.3390/life15091438 (PMC12471357; doi:10.3390/life15091438)
Supplement: Supplementary file 1 [file life-15-01438-s001.zip › life-3846954-supplementary.pdf]

Supplementary Material: Investigating the hemorheological, metabolic and physical performance effect of a core muscle strengthening training program

Tobias Mody, Zsuzsanna Nemethne Gyurcsik, Csaba A. Bakos, Bela Horvath, Barbara Bedocs-Barath, Adam Varga, Adam Attila Matrai, Norbert Nemeth and Sandor Szanto

Table S1. Description of the core training program

**Core Training/Progression**	**1-6 Weeks**	**7-12 Weeks**
Intensity	moderate; 70% of the maximal loading/level	moderate; 70% of the maximal loading/level
Repetition	5 reps/each exercise	5 reps/each exercise
Set	3 X	5 X
Type of muscle contracion	concentric and eccentric, isometric – holding the end position in 5 seconds	concentric and eccentric, isometric – holding the end position in 5 seconds
Aim	Exact implementation of exercises	Progressive training to improve functionality

Table S2. Detailed tasks of the core training program

**Concept of Core Training**	**Effect of Exercises**	**Applied Exercises**
Dynamic Warm-Up	- to apply mobility exercises to improve range of motion (ROM)<br>- to improve performance in physical activities as a result of improved flexibility<br>- to improve blood circulation which limits muscle soreness<br>- to reduce the risk of injury and tissue damage in training<br>- to activate the muscle chains	- leg and arm swings<br>- thoracic mobilization in 4-point-kneeling<br>- walking lizard lunge with thoracic rotation<br>- forward inch worms<br>- cossack squat with hip rotation<br>- crab walk<br>- forward fold and downward dog position
Chest Mobilization	- to activate the local stabilizers which contains the diaphragm<br>- to increase spine ROM<br>- to increase mobility of the rib cage<br>- to improe abdominal-diphragmatic breathing pattern	- to inhale quietly through the nose<br>- to hold the breath for count of 4<br>- to exhale completely but not fastly through the mouth
Targeted Core Strengthening	- to improve and maintain the segmental stability<br>- to protect the pelvic-lumbo-hip complex against load<br>- to increase strenght of outer core muscle contributing the optimal synergist muscle activity and functionality	1. isolated lumbar stabilization - developing conscious muscle activity<br>2. progressive moderate lumbar stabilization integrated into functional exercises in standing and crawling positions<br>- Standing alternate arm or leg raises<br>- 4-point-kneeling alternate arm or leg raises<br>- half-kneeling involving asymmetrical rotational muscle chain activation<br>- deep and high crawling positions<br>- forward and side lunge alternate trunk movements
